# Supplementary material for: Enveloped viruses distinct from HBV induce dissemination of hepatitis D virus in vivo
Source: Nat Commun. 2019 May 8;10:2098. doi: 10.1038/s41467-019-10117-z (PMC6506506; doi:10.1038/s41467-019-10117-z)
Supplement: Supplementary file 1 — Supplementary Information [file 41467_2019_10117_MOESM1_ESM.pdf]

Enveloped viruses distinct from HBV induce dissemination of hepatitis D virus *in vivo*

Authors: Jimena Perez-Vargas[1], Fouzia Amirache[1], Bertrand Boson[1], Chloé Mialon[1], Natalia Freitas[1], Camille Sureau[2], Floriane Fusil[1], and François-Loïc Cosset[1*]

Affiliations :
[1]CIRI – Centre International de Recherche en Infectiologie, Univ Lyon, Université Claude Bernard Lyon 1, Inserm, U1111, CNRS, UMR5308, ENS Lyon, 46 allée d'Italie, F-69007, Lyon, France.
[2]Molecular Virology laboratory, Institut National de la Transfusion Sanguine (INTS), CNRS Inserm U1134, 6 rue Alexandre Cabanel, F-75739 Paris, France.

***Correspondence:** François-Loïc Cosset. CIRI – International Center for Infectiology Research. E-mail: flcosset@ens-lyon.fr

Supplementary Information

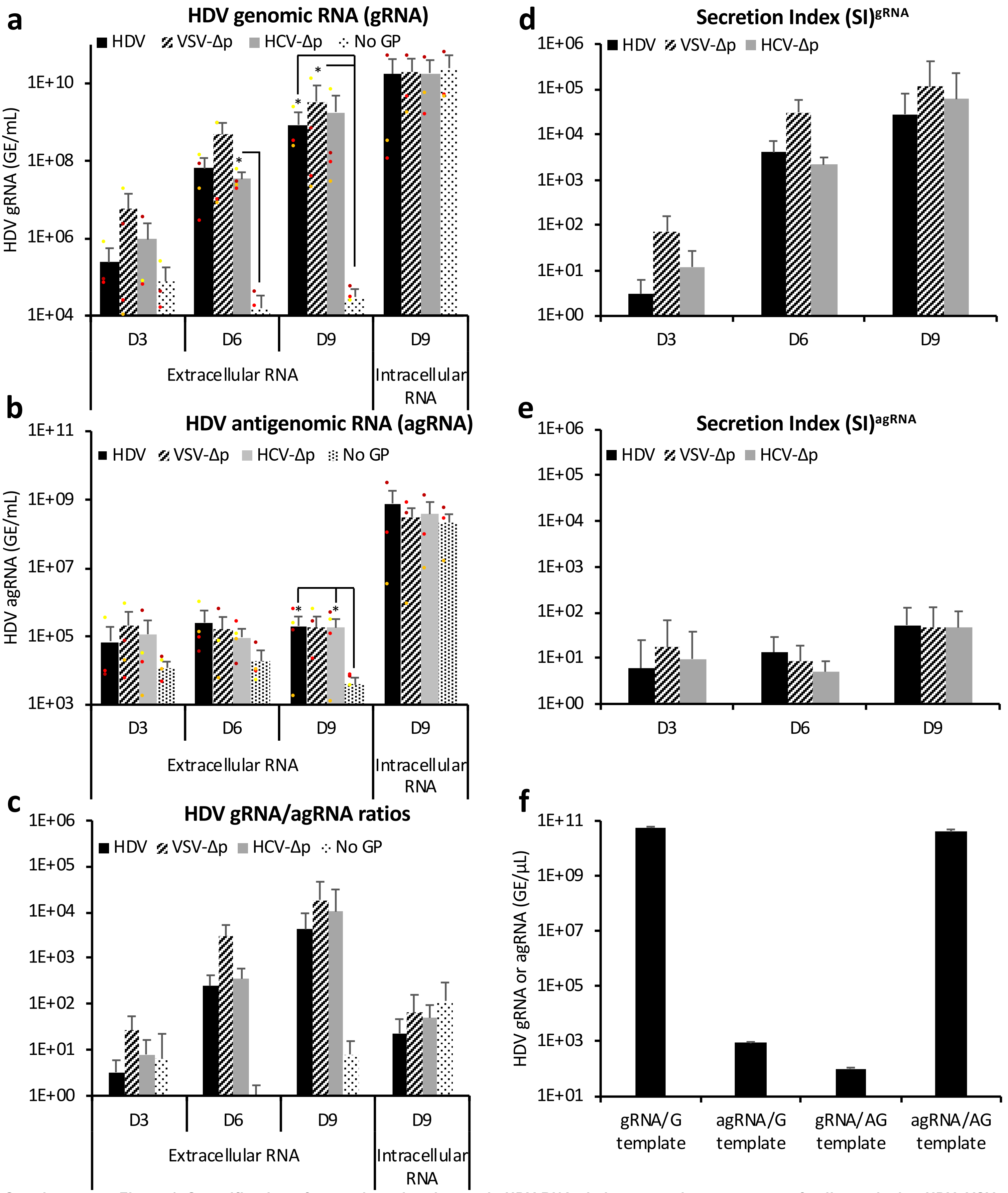

Supplementary Figure 1. Quantification of genomic and antigenomic HDV RNAs in lysates and supernatants of cells producing HDV, VSV-Δp, and HCV-Δp particles. (**a**, **b**) Huh-7 cells were co-transfected with pSVLD3 plasmid coding for HDV RNPs and plasmids coding for either HBV, VSV or HCV surface glycoproteins (GP), resulting in "HDV", "VSV-Δp", and "HCV-Δp" samples, respectively. As control, pSVLD3 was co-transfected with an empty plasmid ("No GP" samples). At day 3, 6 or 9, extracellular HDV RNAs were quantified from cell supernatants by using a strand-specific RTqPCR assay for genomic (gRNA) (**a**) and anti-genomic (agRNA) (**b**) HDV RNAs. Intracellular HDV gRNA and agRNA RNAs were quantified from cell lysates at day 9. HDV RNAs in GE (genome equivalent) are expressed as means (N=4 independent experiments) per mL of cell supernatants for extracellular RNAs or, for intracellular RNAs, per ml of cell lysates containing 10^6 cells. No significant increase of HDV agRNAs over time post-transfection could be detected in the supernatants (**b**), in sharp contrast to the extracellular HDV gRNAs that increased by up to 1,000-fold (**a**). (**c**) The enrichment of HDV gRNAs in secreted particles is reflected by calculating the gRNA/agRNA ratios in HDV, VSV-Δp or HCV-Δp particles from the supernatants and in cell lysates. Particularly, at day 9 post-transfection, the gRNA/agRNA ratios in particles are up to 800-fold higher than the gRNA/agRNA ratios in the cell lysates. (**d, e**) "Secretion Indexes" (SI) were calculated for HDV gRNAs (SI^{gRNA}) (**d**) and for HDV agRNAs (SI^{agRNA}) (**e**) by normalizing extracellular RNAs detected in cell supernatants for HDV, VSV-Δp or HCV-Δp to the "No GP" condition. (**f**) The strand-specific RTqPCR assay allowing quantification of gRNAs or agRNAs was calibrated and validated using either 10^{11} genomic (G) or antigenomic (AG) HDV RNA standards that had been transcribed *in vitro* as templates to detect unspecific quantification. The results indicate specific detection of either template and very low detection of opposite templates. Error bars correspond to standard deviation. Statistical analyses (Student's t-test): $p < 0.05$ (*).

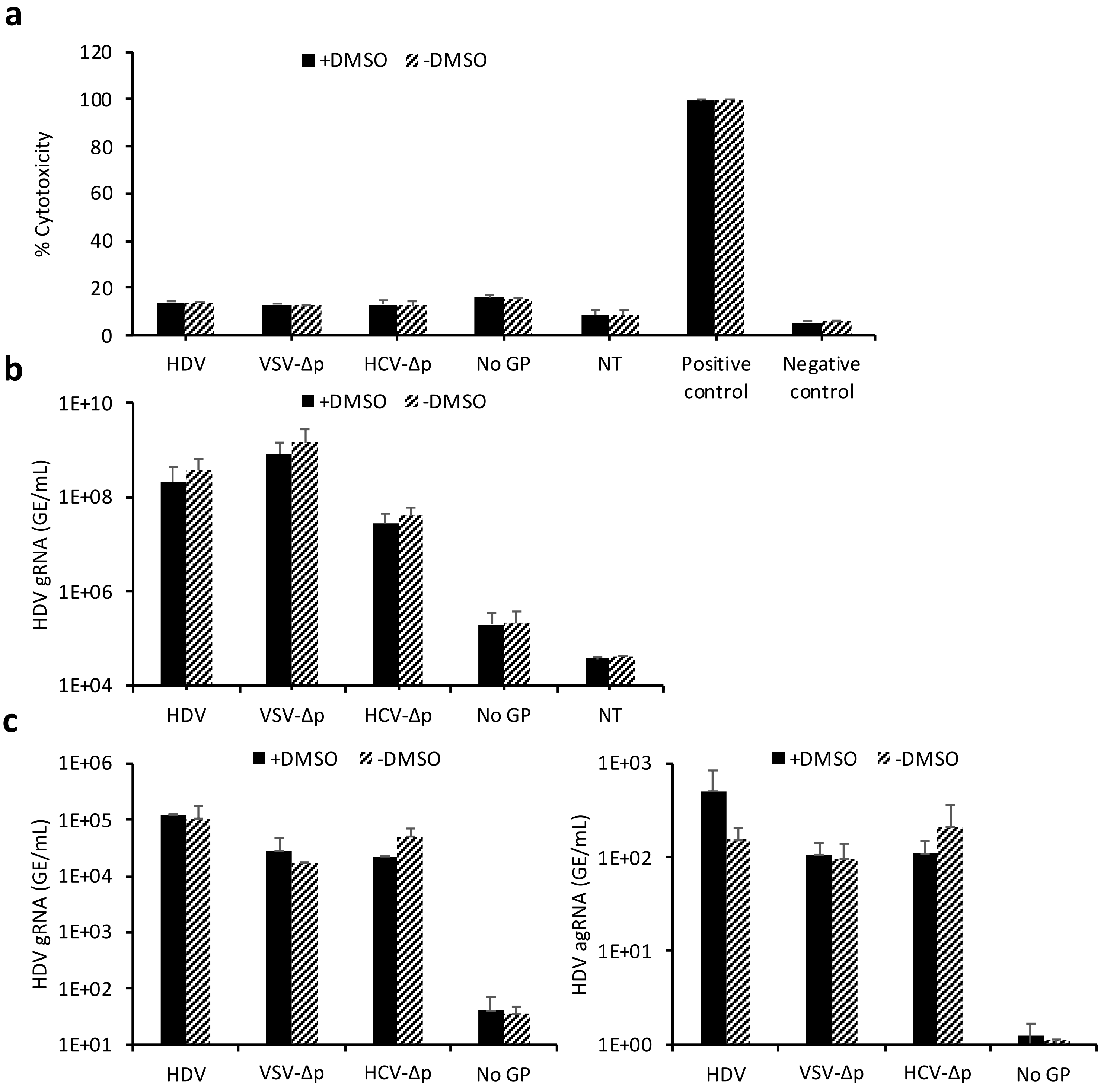

Supplementary Figure 2. DMSO supplementation in producer cell culture media does not affect production of HDV particles. Huh-7 cells were co-transfected with pSVLD3 plasmid coding for HDV RNPs and plasmids coding for either HBV "HDV", VSV "VSV-Δp" or HCV "HCV-Δp" envelope glycoproteins (GP). As control, Huh-7 cells were transfected with pSVLD3 plasmid without envelope proteins (No GP) or were not transfected (NT).(**a-c**) The cells were cultured for 6 days in primary hepatocyte maintenance medium (see Methods section) containing (+DMSO), or not (-DMSO), 2%DMSO to slow cell growth, as indicated. Cell toxicity assessment was performed with the LDH (Pierce Cytotoxicity Assay Kit) using the indicated positive and negative controls of the kit (**a**). The cell supernatants were then filtered and the extracellular RNA was extracted and purified before quantifying HDV gRNAs by strand-specific RTqPCR (**b**). Huh-106 cells were inoculated with the above supernatants. Infected cells were grown for 7 days before total intracellular RNA was purified (**c**). The quantification of intracellular viral nucleic acids in lysates of infected cells were normalized with GAPDH RNAs. The results of HDV gRNAs quantification by strand-specific RTqPCR assays are expressed in GE (genome equivalent) and are displayed as means per mL of cell supernatants for extracellular nucleic acids or, for intracellular nucleic acids, per mL of cell lysates containing 10^6 cells. Source data are provided as a Source Data file. Error bars correspond to standard deviation.

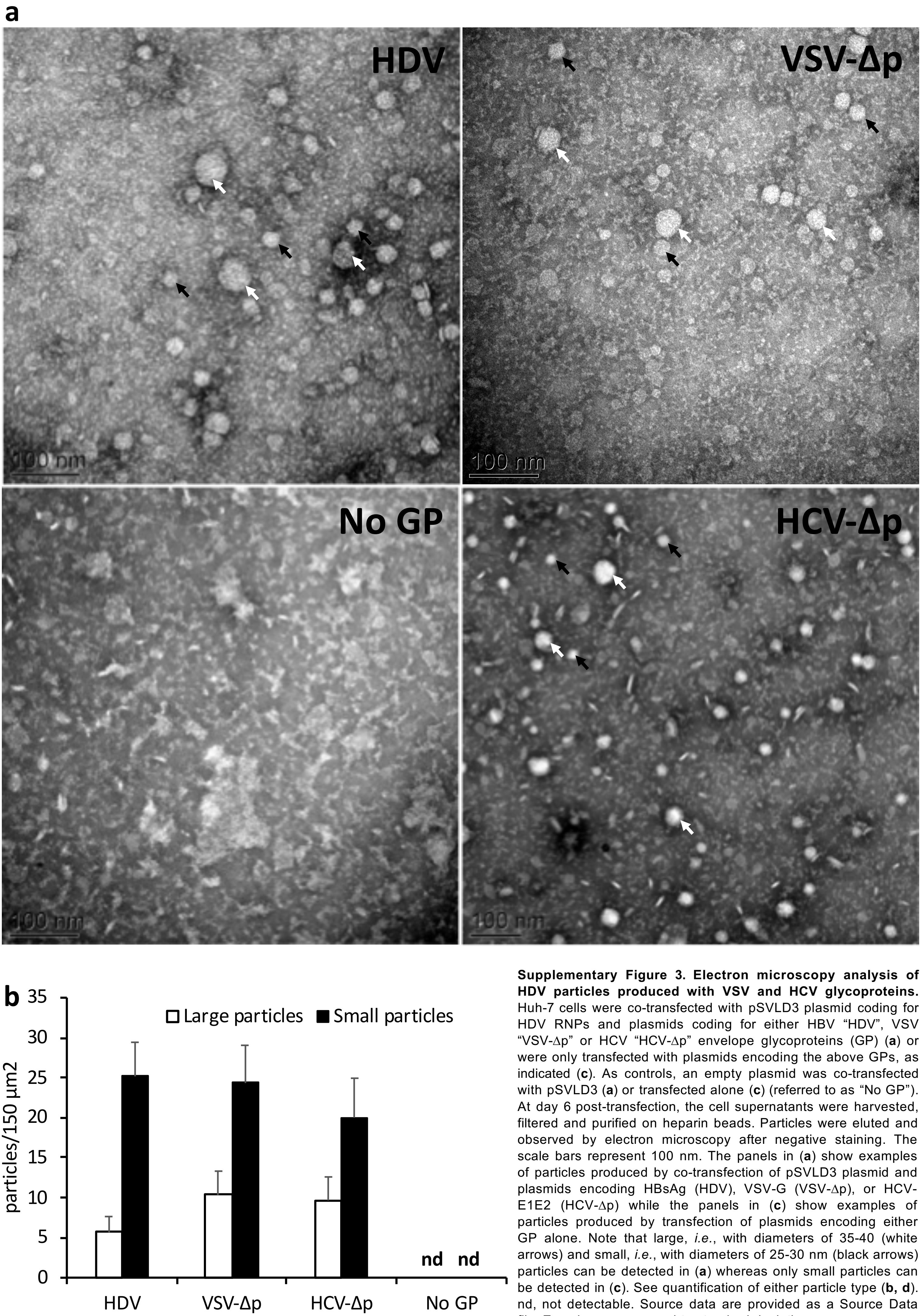

Supplementary Figure 3. Electron microscopy analysis of HDV particles produced with VSV and HCV glycoproteins. Huh-7 cells were co-transfected with pSVLD3 plasmid coding for HDV RNPs and plasmids coding for either HBV "HDV", VSV "VSV-Δp" or HCV "HCV-Δp" envelope glycoproteins (GP) (**a**) or were only transfected with plasmids encoding the above GPs, as indicated (**c**). As controls, an empty plasmid was co-transfected with pSVLD3 (**a**) or transfected alone (**c**) (referred to as "No GP"). At day 6 post-transfection, the cell supernatants were harvested, filtered and purified on heparin beads. Particles were eluted and observed by electron microscopy after negative staining. The scale bars represent 100 nm. The panels in (**a**) show examples of particles produced by co-transfection of pSVLD3 plasmid and plasmids encoding HBsAg (HDV), VSV-G (VSV-Δp), or HCV-E1E2 (HCV-Δp) while the panels in (**c**) show examples of particles produced by transfection of plasmids encoding either GP alone. Note that large, *i.e.*, with diameters of 35-40 (white arrows) and small, *i.e.*, with diameters of 25-30 nm (black arrows) particles can be detected in (**a**) whereas only small particles can be detected in (**c**). See quantification of either particle type (**b, d**). nd, not detectable. Source data are provided as a Source Data file. Error bars correspond to standard deviation.

c

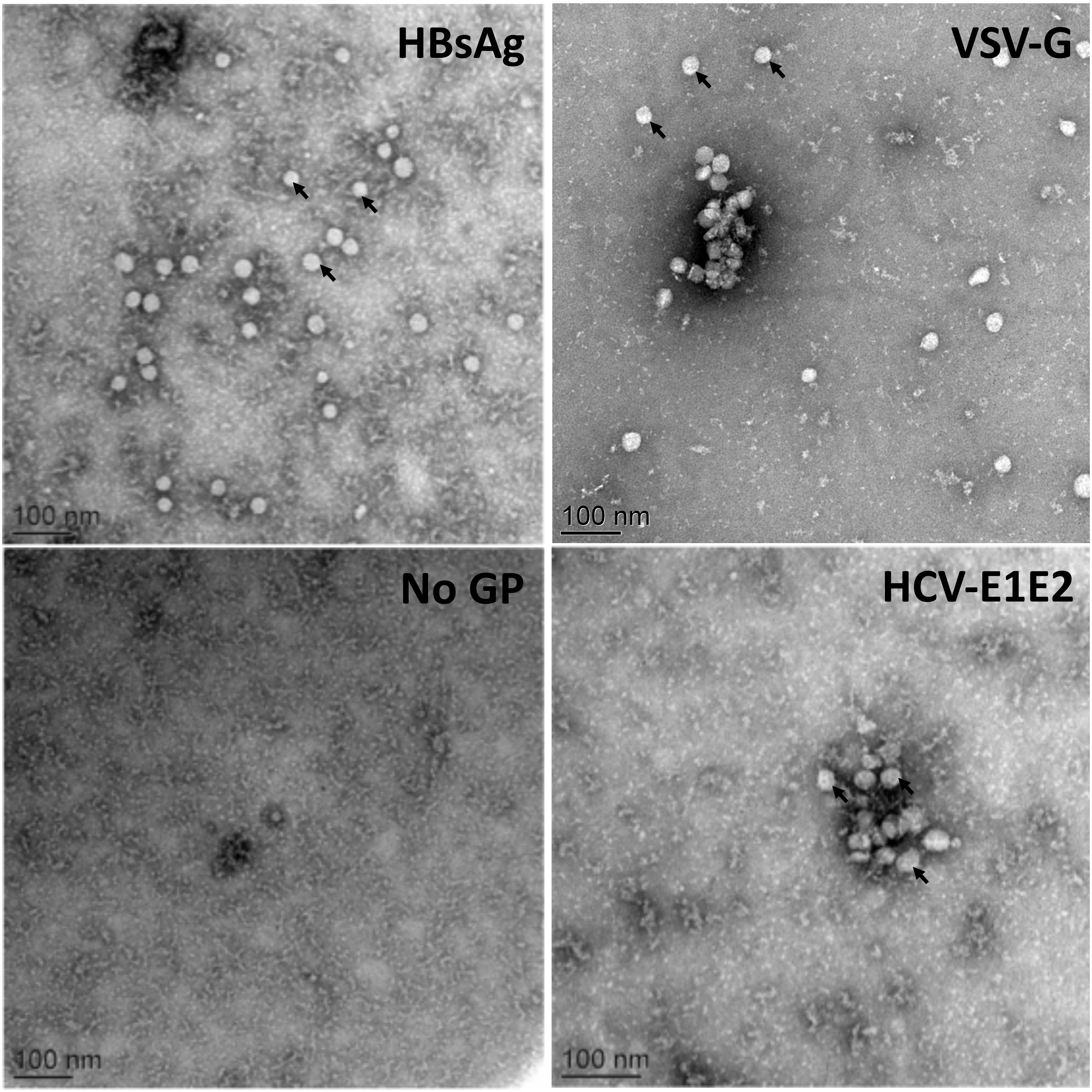

d

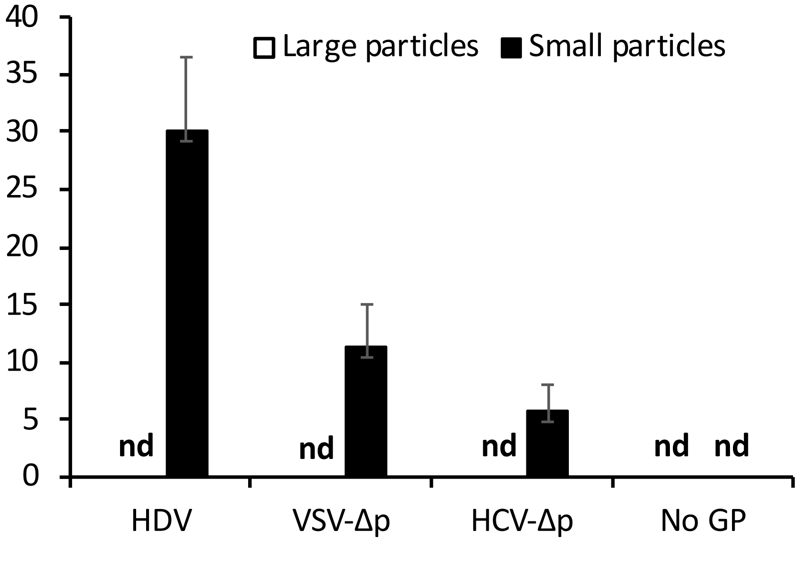

Supplementary Figure 3. Electron microscopy analysis of HDV particles produced with VSV and HCV glycoproteins.

(continued)

a

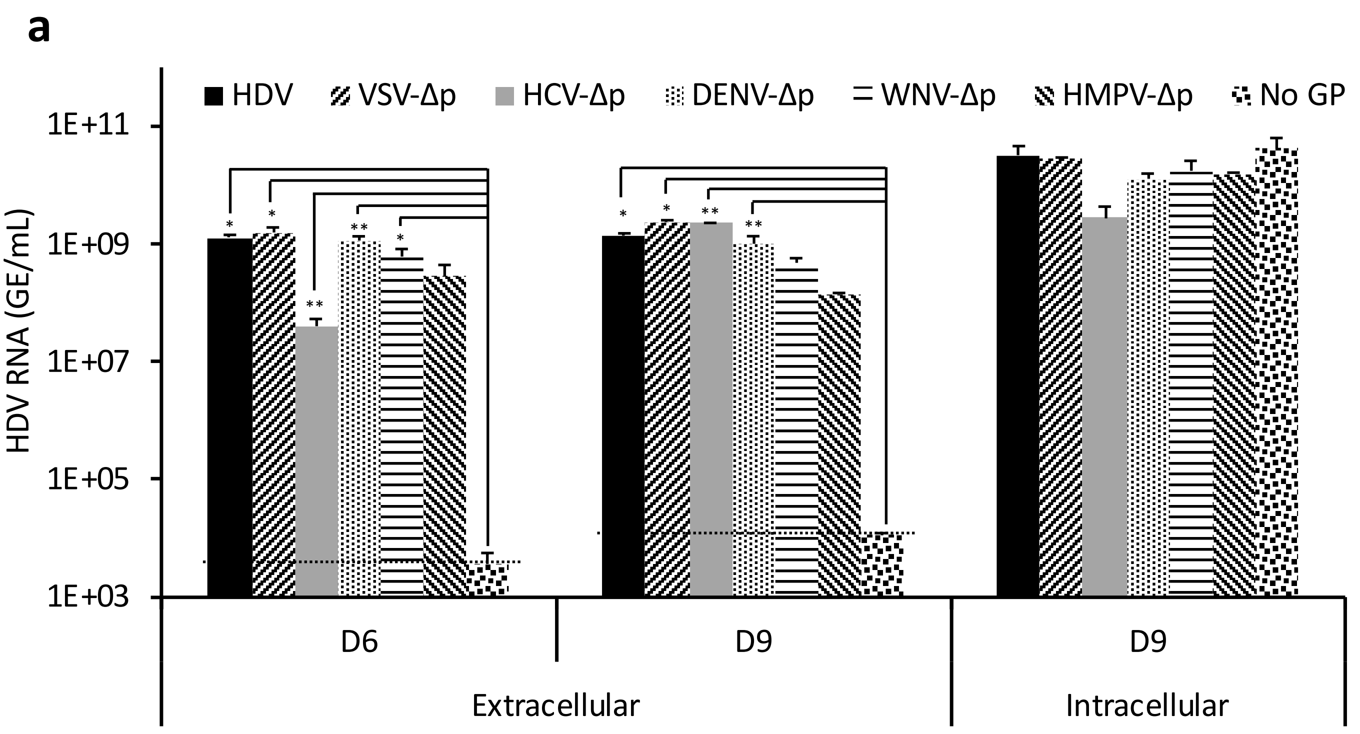

b

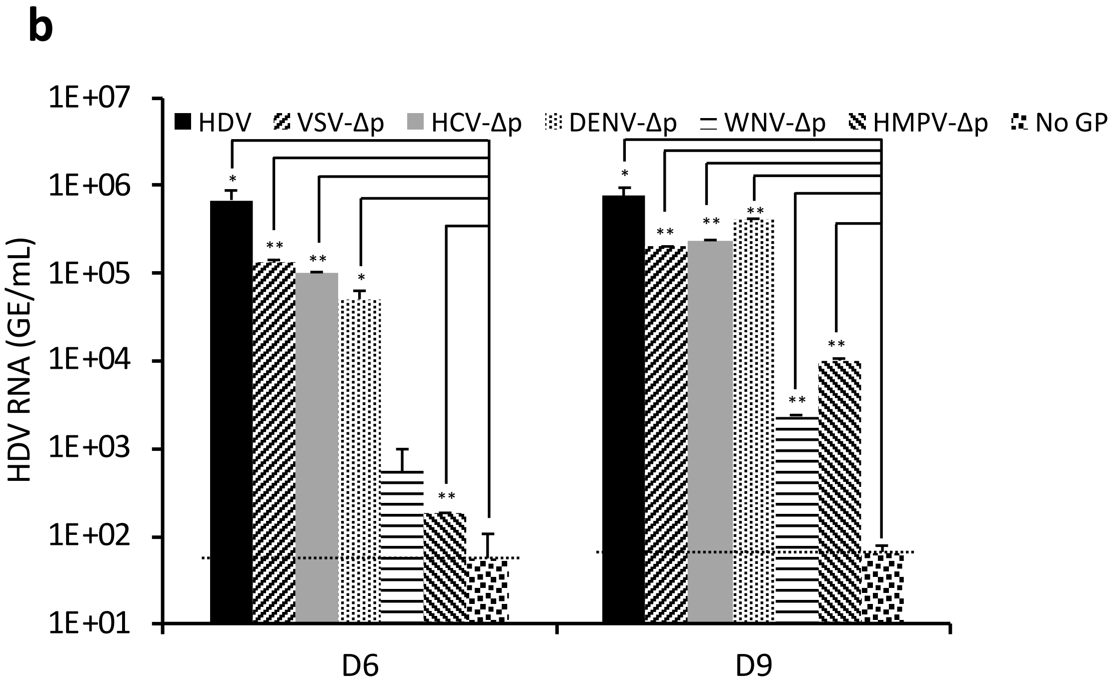

Supplementary Figure 4. Secretion and infectivity of HDV particles enveloped with different GP from 293T cells. (**a**) Cell culture media from 293T cells were harvested at day 6 or 9, as indicated, upon co-transfection of pSVLD3 plasmid coding for HDV RNPs and plasmids coding for HBV (HDV), VSV (VSV-Δp), HCV (HCV-Δp), DENV (DENV-Δp), WNV (WNV-Δp) or HMPV (HMPV-Δp) envelope glycoproteins. As control, pSVLD3 was transfected without envelope proteins (No GP). The cell supernatants were filtered and the extracellular RNA was extracted and purified before quantifying HDV RNAs by RTqPCR. The quantification of intracellular HDV RNAs in cells producing the HDV particles at day 9 post-transfection and normalized with GAPDH RNAs are also shown. HDV RNAs in GE (genome equivalent)/mL are expressed as means (N=5 independent experiments) per ml of cell lysates containing 10^6 cells. (**b**) The infectivity of virus particles present in the cell supernatants that were harvested at day 6 or 9 post-transfection was determined in Huh-106 (NTCP-expressing Huh-7 cells) cells. Infected cells were grown for 7 days before total intracellular RNA was purified. The results of HDV RNAs quantification by RTqPCR are expressed as means after normalization with GAPDH RNAs. The dotted lines represent the experimental thresholds, as defined with the "No GP" controls. Source data are provided as a Source Data file. Error bars correspond to standard deviation. Statistical analyses (Student's t-test): $p < 0.05$ (*); $p < 0.01$ (**).

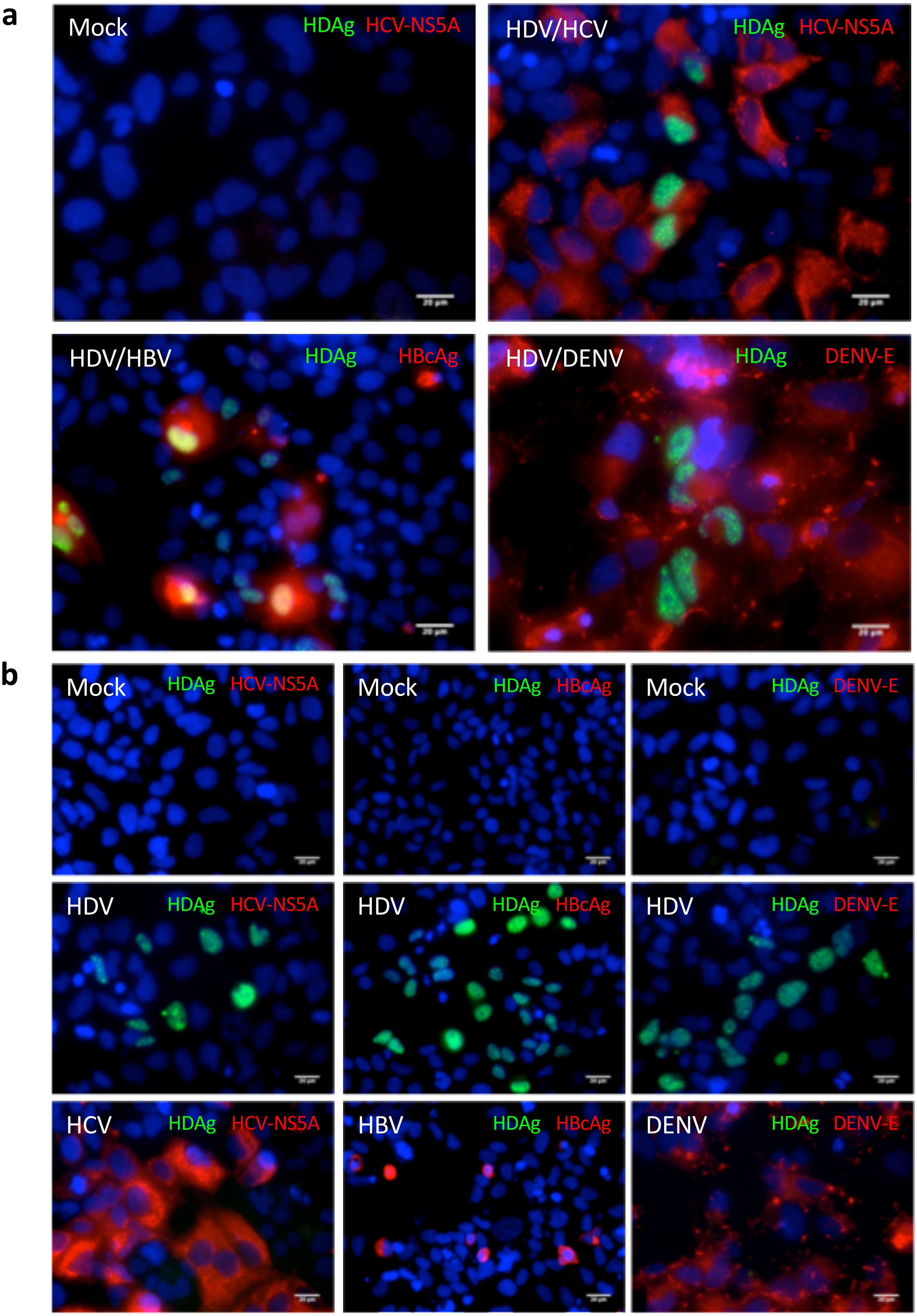

Supplementary Figure 5. Immunofluorescence of HDV RNA-expressing cells superinfected with HBV, HCV and DENV. (**a**) mock-infected cells (Mock) or cells expressing HDV RNAs that were inoculated with live HCV (HDV/HCV), HBV (HDV/HBV) or DENV (HDV/DENV) viruses were fixed, stained for HDAg and HCV-NS5A, HDAg and HBcAg and HDAg and DENV-E, as indicated, counterstained with Hoechst to visualize the nuclei. HDAg (green channel), HCV-NS5A, HBcAg, DENV-E (red channels) and nuclei (blue channel), and were then visualized by immunofluorescence. Scale bars represent 20 µm. (**b**) Controls of immunofluorescence staining. Mock-infected cells (top row), HDV-expressing cells (middle row) and HCV-, HBV- or DENV-infected cells (bottom row) were stained for HDAg (green), nuclei (blue) and HCV-NS5A (red, left column), HBcAg (red, middle column) or DENV-E (red, right column), and visualized by immunofluorescence. Scale bars represent 20 µm.

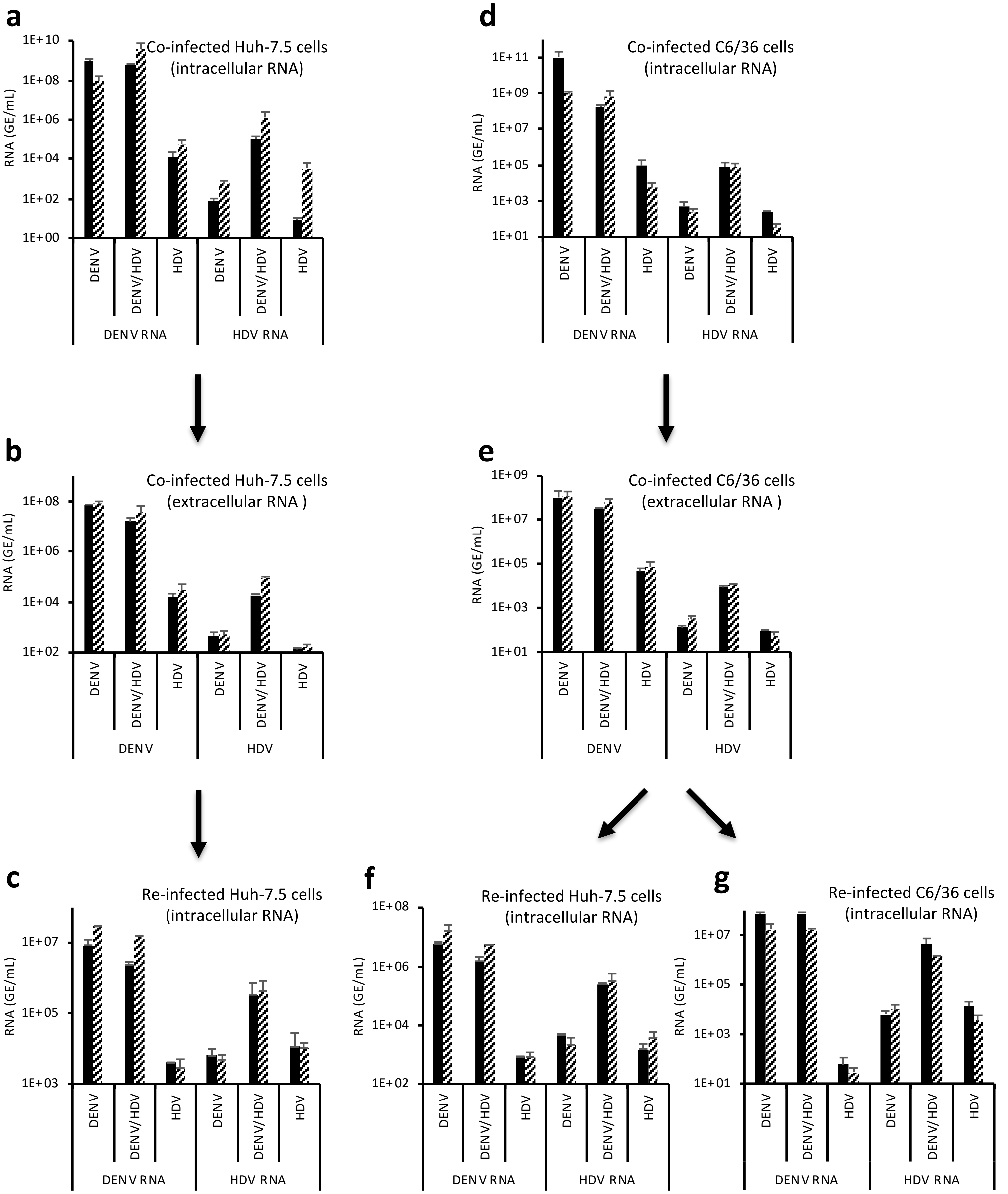

Supplementary Figure 6. HDV/DENV productively infect and propagate in C6/36 mosquito cells. Huh-7.5 (**a**) or C6/36 (**D**) cells were inoculated with low (black bars) *vs.* high (hatched bars) inputs of HDV/DENV (MOI=0.01 and 0.1 FFU/cell for HDV and DENV, respectively) particles, that were purified from HDV/DENV co-infected cells (see Figure 6). Supernatants and lysates from these cells were harvested at day 5 post-infection. The supernatants from HDV/DENV co-infected Huh-7.5 (**b**) or C6/36 (**e**) cells were used, respectively, to re-infect naïve Huh-7.5 (**C**) cells or both Huh-7.5 (**f**) and Huh-106 (**g**) cells. Infection levels were assessed at day 7 post-infection. The nucleic acids present in filtered cell supernatants (**b, e**) as well as in lysates of producer cells (**a, d**) and target cells (**c, f, g**) were extracted and purified for quantification of HDV and DENV RNA by RTqPCR. The quantification of intracellular RNAs in cell lysates were normalized with GAPDH RNAs. The results expressed in GE (genome equivalent) are displayed as means per mL of cell supernatants for RNAs or, for intracellular RNAs, per mL of cell lysates containing 10^6 cells. Source data are provided as a Source Data file. Error bars correspond to standard deviation.

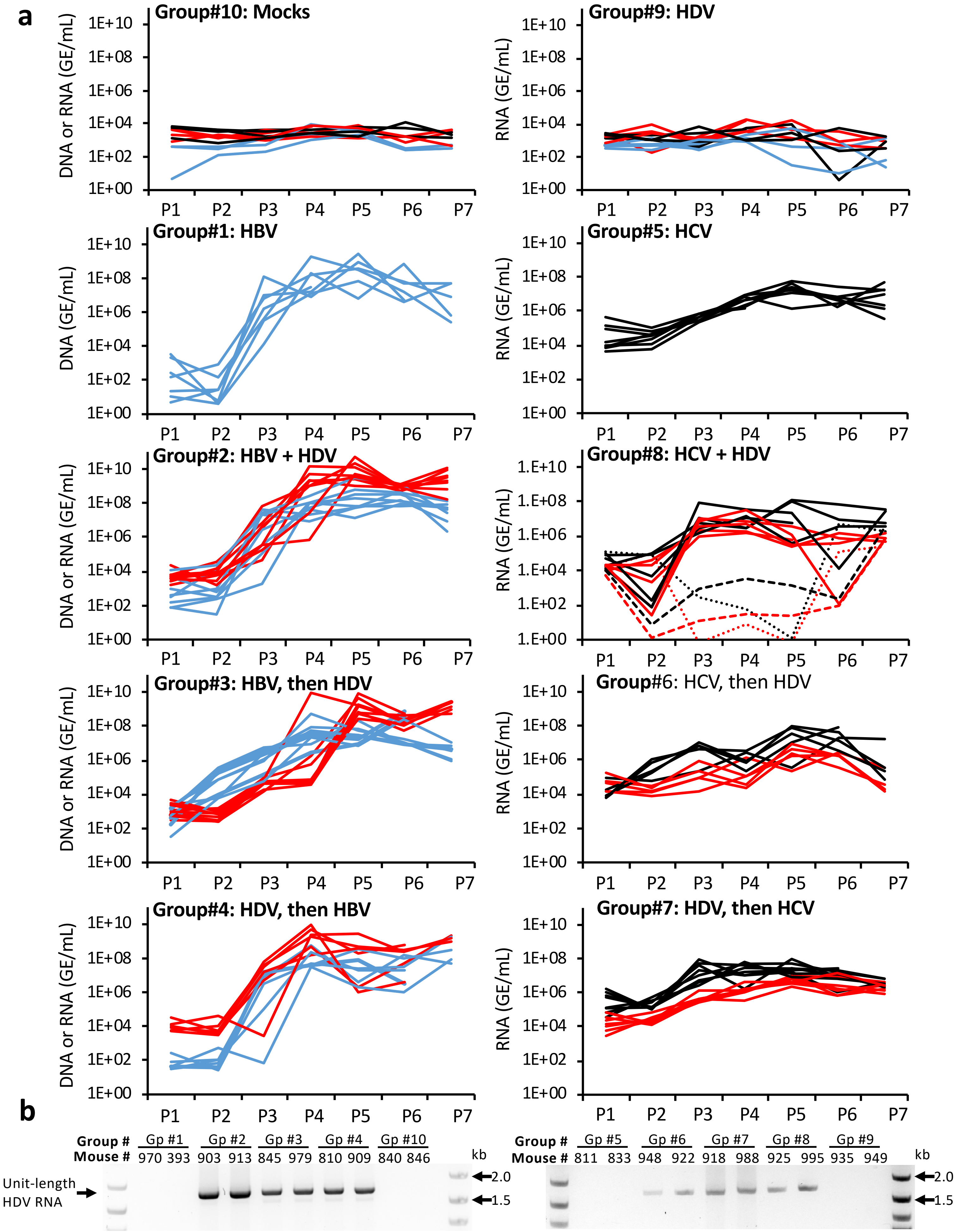

Supplementary Figure 7. HCV propagates HDV particles *in vivo*. NOD-FRG mice, engrafted with primary human hepatocytes (PHH) for two months and displaying HSA levels >15 mg/mL, which corresponded to 40-70% human hepatocytes as assessed by FAH staining (data not shown), were split in different groups that were infected with HDV (10^7 GE/mouse), HBV (10^8 GE/mouse) and/or HCV ($1.5x10^5$ FFU/mouse), as shown in the schedule of Figure 7a. (**a**) At different time points post-infection, blood samples (50 µl) were collected and the viremia in sera was monitored by qPCR on genomes of the indicated viruses (GE/mL of serum). The graphs show the results of viremia of HDV (red lines), HBV (blue lines) and HCV (black lines) in individual mice from all groups as well as from control groups, inoculated with PBS (Mocks; Group#10) or with HDV only (HDV; Group#9). Note the results from two identified mice of Group#8 as displayed in the small (mouse #902) and large (mouse #978) dotted lines that show correlation between HCV and HDV viremia. (**b**) Reverse-transcribed RNAs from sera from co-infected animals were PCR-amplified with HDV-specific primers to reveal the size of transcribed and secreted HDV genomes (HDV RNA unit-length). The results from two mice per group are shown here.

a

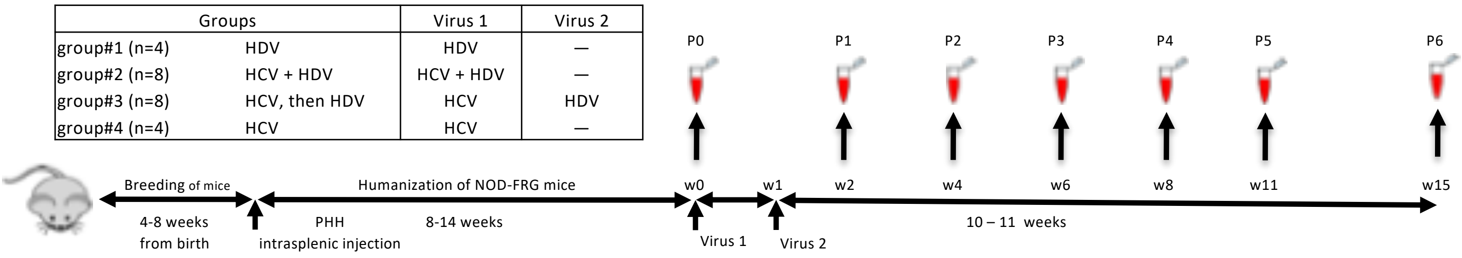

b

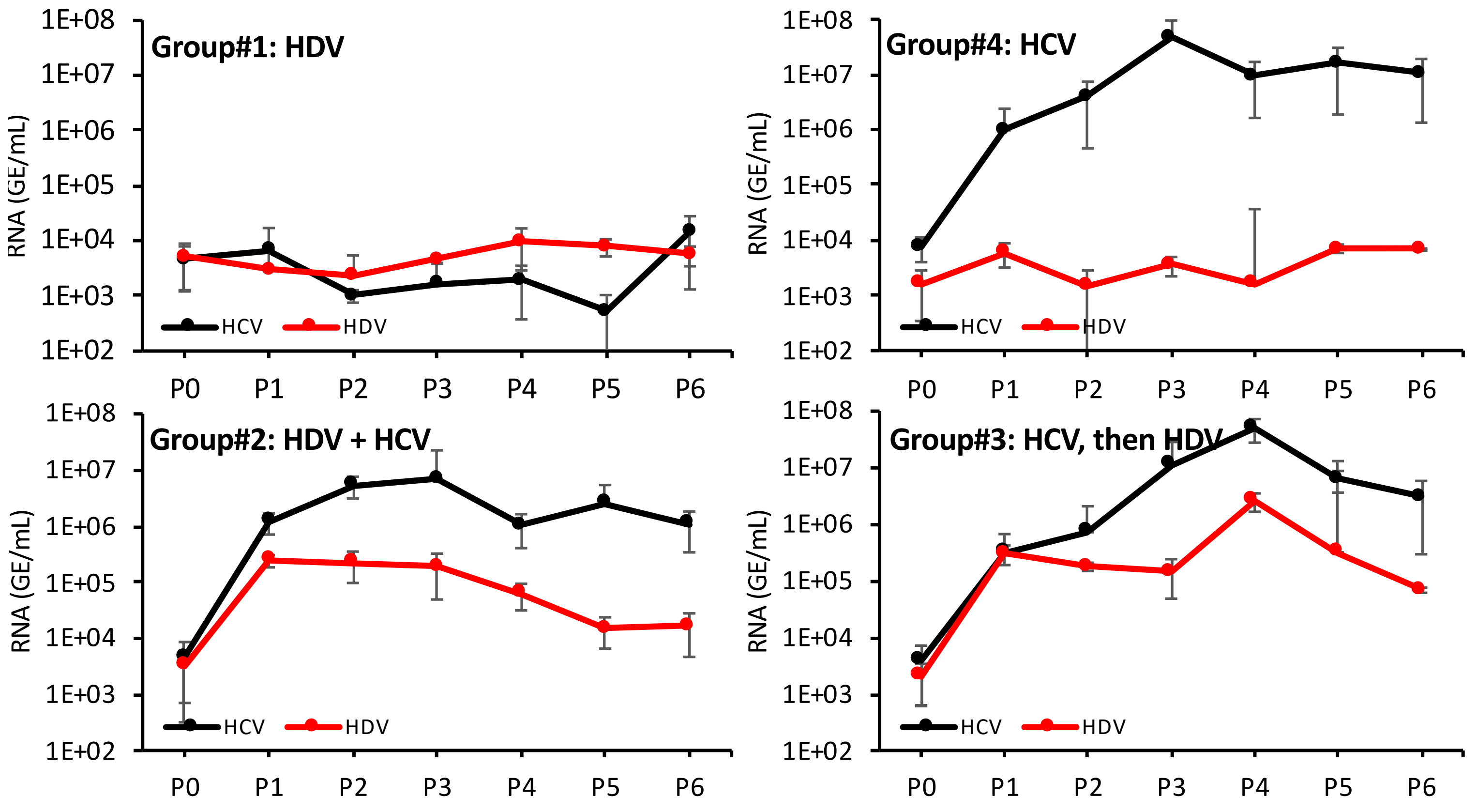

Supplementary Figure 8. 2nd study of HDV propagation *in vivo* by HCV. 4-8 weeks old NOD-FRG mice were engrafted with primary human hepatocytes (PHH). After *ca*. 2-3 months, the animals displaying HSA levels >15 mg/mL were split in 4 groups (N=4 to N=8 independent animals, see Table) that were infected with HDV (10^7 GE/mouse) and/or HCV ($1.5x10^5$ FFU/mouse), as shown in the schedule (**a**). At different time points post-infection, blood samples (50 µl) were collected and the viremia in sera was monitored by qPCR on the genomes of the indicated viruses (GE/mL of serum) (**b**). The graphs show the mean results of viremia of HDV (red lines) and HCV (black lines). Source data are provided as a Source Data file. Error bars correspond to standard deviation.
