# Supplementary material for: Enveloped viruses distinct from HBV induce dissemination of hepatitis D virus in vivo
Source: Nat Commun. 2019 May 8;10:2098. doi: 10.1038/s41467-019-10117-z (PMC6506506; doi:10.1038/s41467-019-10117-z)
Supplement: Supplementary file 3 — Reporting Summary [file 41467_2019_10117_MOESM3_ESM.pdf]

nature research

Corresponding author(s): François-Loic COSSET

Last updated by author(s): Apr 13, 2019

Reporting Summary

Nature Research wishes to improve the reproducibility of the work that we publish. This form provides structure for consistency and transparency in reporting. For further information on Nature Research policies, see Authors & Referees and the Editorial Policy Checklist.

Statistics

For all statistical analyses, confirm that the following items are present in the figure legend, table legend, main text, or Methods section.

n/a	Confirmed	
☐	☒	The exact sample size (*n*) for each experimental group/condition, given as a discrete number and unit of measurement
☐	☒	A statement on whether measurements were taken from distinct samples or whether the same sample was measured repeatedly
☐	☒	The statistical test(s) used AND whether they are one- or two-sided<br>*Only common tests should be described solely by name; describe more complex techniques in the Methods section.*
☒	☐	A description of all covariates tested
☒	☐	A description of any assumptions or corrections, such as tests of normality and adjustment for multiple comparisons
☐	☒	A full description of the statistical parameters including central tendency (e.g. means) or other basic estimates (e.g. regression coefficient) AND variation (e.g. standard deviation) or associated estimates of uncertainty (e.g. confidence intervals)
☐	☒	For null hypothesis testing, the test statistic (e.g. *F*, *t*, *r*) with confidence intervals, effect sizes, degrees of freedom and *P* value noted<br>*Give P values as exact values whenever suitable.*
☒	☐	For Bayesian analysis, information on the choice of priors and Markov chain Monte Carlo settings
☒	☐	For hierarchical and complex designs, identification of the appropriate level for tests and full reporting of outcomes
☒	☐	Estimates of effect sizes (e.g. Cohen's *d*, Pearson's *r*), indicating how they were calculated

Our web collection on statistics for biologists contains articles on many of the points above.

Software and code

Policy information about availability of computer code

Data collection	*Provide a description of all commercial, open source and custom code used to collect the data in this study, specifying the version used OR state that no software was used.*
Data analysis	*Provide a description of all commercial, open source and custom code used to analyse the data in this study, specifying the version used OR state that no software was used.*

For manuscripts utilizing custom algorithms or software that are central to the research but not yet described in published literature, software must be made available to editors/reviewers. We strongly encourage code deposition in a community repository (e.g. GitHub). See the Nature Research guidelines for submitting code & software for further information.

Data

Policy information about availability of data

All manuscripts must include a data availability statement. This statement should provide the following information, where applicable:

- Accession codes, unique identifiers, or web links for publicly available datasets
- A list of figures that have associated raw data
- A description of any restrictions on data availability

The datasets generated during the current study are available from the corresponding author upon reasonable request. The source data underlying figures and Supplementary Figures are provided as a Source Data file.

Field-specific reporting

Please select the one below that is the best fit for your research. If you are not sure, read the appropriate sections before making your selection.

☒ Life sciences ☐ Behavioural & social sciences ☐ Ecological, evolutionary & environmental sciences

For a reference copy of the document with all sections, see nature.com/documents/nr-reporting-summary-flat.pdf

Life sciences study design

All studies must disclose on these points even when the disclosure is negative.

Sample size	No sample size calculation was performed. Most of our experiments involved testing infection in cultured cells where sample size is not relevant. Experiments involving animals did not require statistical analysis of the results as we were mainly testing for the ability of HCV to propagates HDV in vivo. We therefore did not perform any sample size calculation.
Data exclusions	We did not exclude any data in any of the experiments performed in cultured cells.
Replication	Most experiments performed in cultured cells were performed in two to five independent biological replicates.<br><br>Experiments involving propagation of HDV particles in FRG mice (Figure 7 and S7) were performed in 2 independent biological replicates, the first consisting in the injection of 65 mice (4 controls groups with 4 or 8 mice and 6 groups with different virus combination of 8 mice each) for the first replicate and 24 mice for the second biological replicate (2 controls groups of 4 mice and 2 groups of different virus combination with 8 mice each). Again, we were looking for a propagation results so the number of animals used in each experiment was mainly defined by their availability.
Randomization	Our experiments did not involve testing on a large number of individuals or groups of individuals. As such, randomization of samples is not relevant to our study.
Blinding	For all animal experiments, the persons extracting and quantifying RNA were not blinded to mice groups during experiments. Data reported for mouse experiments are not subjective but rather based on quantitative RTqPCR from serum samples.

Reporting for specific materials, systems and methods

We require information from authors about some types of materials, experimental systems and methods used in many studies. Here, indicate whether each material, system or method listed is relevant to your study. If you are not sure if a list item applies to your research, read the appropriate section before selecting a response.

Materials & experimental systems

n/a	Involved in the study
☐	☒ Antibodies
☐	☒ Eukaryotic cell lines
☒	☐ Palaeontology
☐	☒ Animals and other organisms
☒	☐ Human research participants
☒	☐ Clinical data

Methods

n/a	Involved in the study
☒	☐ ChIP-seq
☒	☐ Flow cytometry
☒	☐ MRI-based neuroimaging

Antibodies

Antibodies used	Anti-HDAg SE1679 rabbit polyclonal antibody.<br>Anti-E2 AR3A (kind gift from M. Law)<br>Anti-HBsAg Hs33 (GeneTex, Cat no. GTX41723)<br>Anti-CD81 JS-81 (BD Pharmingen, Cat no. 555675)<br>Anti-LDLr C7 (Santa Cruz Biotechnology, Cat no. sc-18823)<br>Anti-DENV-E 3H5 (kind gift from P. Desprès)<br>Anti-NS5A 9E10 (kind gift of C. Rice)<br>Anti-HBcAg (C. Sureau)
Validation	Anti HDAg SE1679 rabbit polyclonal serum validation in Verrier ER, et al. A targeted functional RNA interference screen uncovers glypican 5 as an entry factor for hepatitis B and D viruses. Hepatology 63, 35-48 (2016).<br>Anti-E2 AR3A mAb: validation in Law M, et al. Broadly neutralizing antibodies protect against hepatitis C virus quasispecies challenge. Nat Med 14, 25-27 (2008).<br>Anti-CD81 JS-81 mAb (BD Pharmingen, Cat no. 555675): validation in Meuleman P, et al. Anti-CD81 antibodies can prevent a Hepatitis C virus infection in vivo. Hepatology 48, 1761-1768 (2008).<br>Anti-LDLr C7 mAb (Santa Cruz Biotechnology, Cat no. sc-18823): validation in Albecka A, et al. Role of low-density lipoprotein

receptor in the hepatitis C virus life cycle. Hepatology 55, 998-1007 (2012).
Anti-DENV-E 3H5 mAb (kind gift from P. Desprès): validation in Costin JM, et al. Mechanistic Study of Broadly Neutralizing Human Monoclonal Antibodies against Dengue Virus That Target the Fusion Loop. J Virol 87, 52-67 (2013). December E, et al. Sensing of Immature Particles Produced by Dengue Virus Infected Cells Induces an Antiviral Response by Plasmacytoid Dendritic Cells. Plos Path 10, e1004434 (2014).
Anti-NS5A 9E10 mAb (kind gift of C. Rice): validation in Boson B, et al. Daclatasvir Prevents Hepatitis C Virus Infectivity by Blocking Transfer of the Viral Genome to Assembly Sites. Gastroenterology 152, 895-907 (2017).
Anti-HBsAg Hs33 mAb (GeneTex, Cat no. GTX41723): validation is this work Figure 3A.
Anti-HBcAg human serum: validation in this work Figure 6H and Supplemental Figure 5.

Eukaryotic cell lines

Policy information about cell lines

Cell line source(s)

The HEK293 cells was purchased from ATCC CRL-1573.
The C6/36 cells was purchased from ATCC CRL-1660.
The Huh-7.5 cells was a kind gift of C. Rice.
The Huh-106 cells were generated from Huh-7 cells by C. Sureau (Blanchet M et al. 2014).

Authentication

The Huh-106 cells were tested for the expression of NTCP at the cell surface by flow cytometry.

Mycoplasma contamination

All immortalized cell lines used in this study were tested negative for mycoplasma contamination using a commercial kit (MycoAlert™ Mycoplasma Detection Kit, catalog number: LT07).

Commonly misidentified lines
(See ICLAC register)

None of the cell lines used in our study are listed in the ICLAC database of commonly misidentified cell lines.

Animals and other organisms

Policy information about studies involving animals; ARRIVE guidelines recommended for reporting animal research

Laboratory animals

Mouse, NOD FRG mice (Fah-/-/Rag2-/-/Il2rg-/-) (Yecuris corporation), female, 12 weeks old.

Wild animals

The study did not involve the use of wild animals.

Field-collected samples

The study did not involve samples collected from the field.

Ethics oversight

Authorization Agreement C2EA-15, “Comité Rhône-Alpes d’Ethique pour l’Expérimentation Animale”, Lyon, France - APAFIS#1570-2015073112163780

Note that full information on the approval of the study protocol must also be provided in the manuscript.
